# Pumping iron, keeping FIT: How MYB30 regulates FIT stability during plant iron deficiency

**DOI:** 10.1093/plcell/koaf134

**Published:** 2025-05-22

**Authors:** Linhan Sun

**Affiliations:** Assistant Features Editor, The Plant Cell, American Society of Plant Biologists; Department of Biochemistry and Molecular Biology, The Pennsylvania State University, University Park, PA 16802, USA

The well-known cartoon character Popeye eats spinach for its high iron (Fe) content, which gives him the strength to deal with his nemesis, Brutus. Plants also rely on this critical element to grow properly. In the model plant Arabidopsis (*A. thaliana*), a pivotal transcription factor FER-LIKE IRON DEFICIENCY-INDUCED TRANSCRIPTION FACTOR (FIT) helps plants respond to Fe deficiency by inducing the expression of iron transporter genes, including *FERRIC REDUCTASE OXIDASE2* (*FRO2*) and *IRON TRANSPORTER1* (*IRT1*; Colangelo and Guerinot 2004). When Fe stores are replenished, 2 BRUTUS-LIKE E3 ubiquitin ligases, BTSL1 and BTSL2, mediate the ubiquitination and degradation of FIT, thus preventing excessive iron uptake (Rodríguez-Celma et al. 2019). Surprisingly, even under Fe deficiency, the expression of *BTSL1* and *BTSL2* also increases (Rodríguez-Celma et al. 2019). This paradox raises the question of how FIT escapes the fate of being degraded under Fe deficiency, when it is needed the most.

In new work, Hongyun Zhao and colleagues (Zhao et al. 2025) reveal a role for another transcription factor, MYB30, in the stabilization of FIT during Fe deficiency. First, they observed that 2 *MYB30* T-DNA insertion loss-of-function mutants (*myb30-1* and *myb30-2*) exhibited more severe Fe deficiency phenotypes, relative to the wild type, such as stronger inhibition of root elongation and diminished expression of *FRO2* and *IRT1*. Conversely, the overexpression lines (OX-#1 and OX-#2) showed enhanced resistance to Fe deficiency stress and elevated levels of *FRO2* and *IRT1*. The mRNA transcript and protein levels of MYB30 were also significantly upregulated under Fe deficiency. These results pointed to the role of MYB30 as a positive regulator of Fe deficiency. To further explore how MYB30 is involved in this process and identify other regulators that work in concert with MYB30, the authors performed a yeast 2-hybrid screening of MYB30-interacting proteins and identified BTSL1 and BTSL2 as candidate interacting partners. Using protein-protein interaction assays, the authors further confirmed that the N-terminus of MYB30 (containing its DNA-binding domain) interacted with the C terminus region of BTSL1. The overlapping expression patterns of MYB30 and BTSL1/2 at the root suggested that MYB30 functions in concert with BTSL1/2 in the Fe-deficiency pathway.

Counterintuitively, the authors discovered that these 2 E3 ubiquitin ligases did not appear to mediate the degradation of MYB30 itself, indicating an unconventional link between BTSL1/2 and MYB30. Given the well-established interaction between BTSL1/2 and FIT (Rodríguez-Celma et al. 2019), the authors hypothesized that the missing link between BTSL1/2 and MYB30 could be FIT. Indeed, the authors discovered that FIT interacted with MYB30 both in yeast and in planta and that the N-terminal DNA-binding region of MYB30 alone could physically interact with FIT. The authors used a series of biochemical experiments to demonstrate that the absence of MYB30 resulted in accelerated degradation of FIT under Fe deficiency, while overexpression of MYB30 exhibited the opposite effects on FIT stability. More importantly, using in vivo co-immunoprecipitation experiments in both tobacco leaves and Arabidopsis protoplasts, they showed that the addition of MYB30 reduced the strength of the interaction between BTSL1 and FIT, and overexpression of MYB30 also curtailed the ubiquitination and degradation of FIT mediated by BTSL1. Collectively, these data suggested that MYB30 functions to interfere with the interaction between BTSL proteins and FIT, thus stabilizing FIT under iron deficiency conditions.

In this comprehensive work, Zhao et al. (2025) uncovered an exciting mechanism by which Arabidopsis responds to Fe deficiency. This response resembles the textbook “competitive inhibition” mechanism: When Fe levels are sufficient, BTSL targets FIT for degradation. However, when Fe levels drop, MYB30 functions as a “decoy” for BTSL1/2, protecting FIT from being degraded by BTSL1/2 under Fe-deficiency. Fine-tuning FIT protein levels and ensuring proper cellular responses mediated by FIT is at the heart of this response (Fig.). This work also opens the door for exploring similar mechanisms regulating the stability of other transcription factors, for example, other key bHLH transcription factors involved in the Fe-deficiency response that are targeted by other E3 ligases (Selote et al. 2015). When plants “pump iron,” they might be able to combat BRUTUS through different mechanisms but with similar underlying themes.

**Figure.**
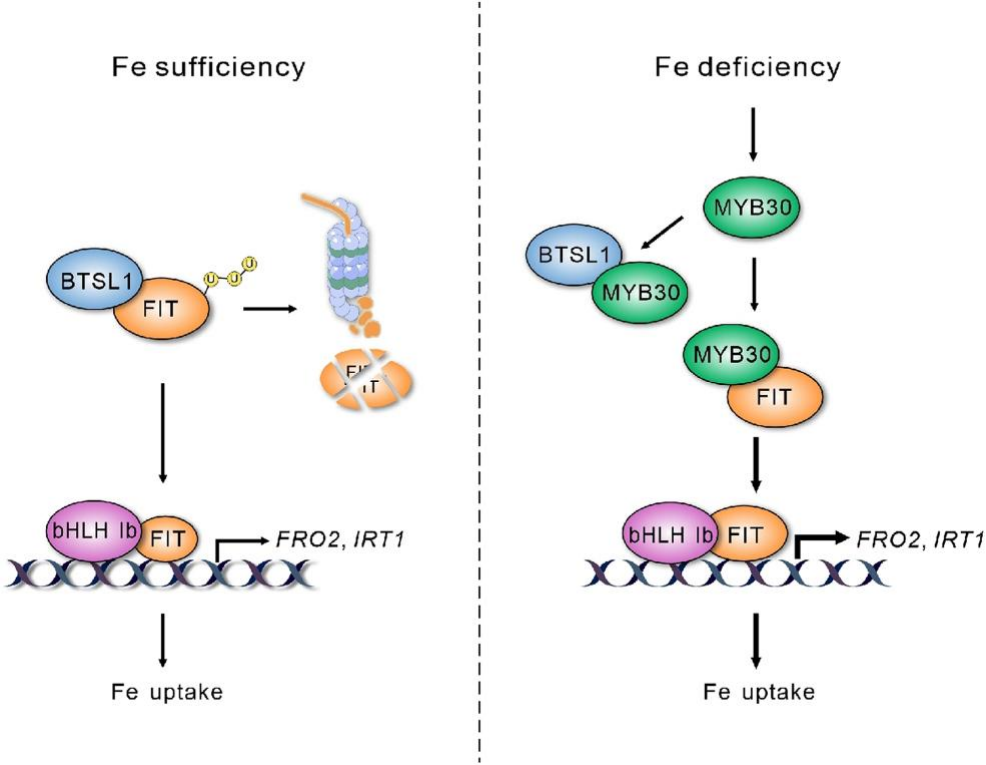
Model of the MYB30-FIT-BTSL1 module and how it functions to maintain Fe homeostasis. Under Fe sufficiency (left), BTSL1 mediates the proteasomal degradation of FIT, which in turn prevents the excessive uptake of Fe. Under Fe deficiency (right), MYB30 stabilizes FIT by interfering with FIT-BTSL1 interaction, thus promoting the expression of FIT-regulated iron transporter genes (*FRO2* and *IRT1*) and Fe uptake. Reprinted from Zhao et al. (2025), Figure 8.

## Recent related articles in *The Plant Cell*:

- Zhao et al. (2024) reported that MYB30 also modulates nitric oxide (NO)-induced seed germination in Arabidopsis.
- Paffrath et al. (2024) showed that a group of secondary metabolites called coumarins is involved in the Fe reduction process in roots mediated by FERRIC REDUCTION OXIDASE2 (FRO2).
- Chia et al. (2023) reported on Arabidopsis OLIGOPEPTIDE TRANSPORTER 3 (OPT3), a Fe transporter localized in the phloem, that mediates systemic responses to both Fe deficiency and copper (Cu) deficiency, highlighting the complex crosstalk between Fe and Cu homeostasis.

## Dive Curated Terms

The following phenotypic, genotypic, and functional terms are of significance to the work described in this paper:

MYB30 Gramene: AT3G28910
MYB30 Araport: AT3G28910

IRT1 Gramene: AT4G19690
IRT1 Araport: AT4G19690

BTSL1 Gramene: AT1G74770
BTSL1 Araport: AT1G74770

BTSL2 Gramene: AT1G18910
BTSL2 Araport: AT1G18910

FRO2 Gramene: At1G01580
FRO2 Araport: At1G01580
